# Acute and Chronic Effects of Aerobic Exercise on Motor Inhibition and Cortical Excitability in Adolescents With Attention Deficit Hyperactivity Disorder

**DOI:** 10.1002/brb3.71600

**Published:** 2026-08-05

**Authors:** Jia‐Ling Sun, Jung‐Chi Chang, Pou‐Leng Cheong, Hsiao‐I Kuo

**Affiliations:** ^1^ School and Graduate Institute of Physical Therapy National Taiwan University Taipei Taiwan; ^2^ Department of Psychiatry National Taiwan University Hospital Taipei Taiwan; ^3^ Department of Pediatrics National Taiwan University Hospital Hsin‐Chu Hospital Hsinchu Taiwan; ^4^ Department of Biological Science and Technology National Yang Ming Chiao Tung University Hsinchu Taiwan

**Keywords:** adolescents, aerobic exercise, attention deficit hyperactivity disorder, cortical excitability, motor inhibition

## Abstract

**Introduction:**

Accumulating evidence suggests that acute aerobic exercise facilitates improvement in motor inhibition among adolescents with attention‐deficit hyperactivity disorder (ADHD), potentially through exercise‐induced modulation of cortical excitability. The present study aimed to investigate the effects of both acute and chronic aerobic exercise on motor inhibition and cortical excitability, and to elucidate the relationship between these variables in adolescents diagnosed with ADHD.

**Methods:**

A partial cross‐over experimental design was employed. Twenty‐four adolescents with ADHD underwent assessments of motor inhibition and cortical excitability before and after engaging in either acute or chronic aerobic exercise, or a control intervention.

**Results:**

Both acute and chronic aerobic exercise elicited significant enhancements in motor inhibition, with chronic aerobic exercise demonstrating more pronounced effects. Furthermore, chronic aerobic exercise resulted in a significant reduction in cortical excitability compared to both the control condition and acute aerobic exercise. In addition, the degree of improvement in motor inhibition was positively associated with increased cortical inhibition.

**Discussion:**

These findings suggest that the enhancement of motor inhibition following aerobic exercise in adolescents with ADHD may be underpinned by specific neurophysiological mechanisms related to cortical excitability. Particularly, chronic aerobic exercise appears to exert a more robust modulatory effect, highlighting its potential as a therapeutic strategy for improving motor inhibitory function in this population.

## Graphical abstract

In adolescents with ADHD, acute and chronic aerobic exercise enhance motor inhibition. Chronic exercise yields greater benefits and reduces cortical excitability, linking behavioral gains to neurophysiological changes. These findings support sustained exercise as a therapeutic strategy for improving inhibitory control.

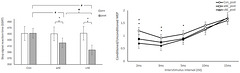

## 1 Introduction

Adolescence represents a pivotal stage of human development, characterized by profound physiological, psychological, and social transformations (Polanczyk et al. 2007). During this period, individuals are increasingly confronted with multifaceted challenges, including evolving social dynamics, escalating academic demands, and the progressive transition toward adult roles and responsibilities (Polanczyk et al. 2007). Among adolescents, approximately 3%–7% are affected by attention‐deficit hyperactivity disorder (ADHD), a condition recognized as the most prevalent neuropsychiatric disorder in this age group globally (Polanczyk et al. 2007; Kessler et al. 2005). ADHD is defined by a persistent pattern of inattention and/or hyperactivity‐impulsivity that interferes with functioning or development (Arnsten 2006).

The pathophysiology of ADHD is complex and multifactorial, encompassing both neurophysiological and neurochemical alterations (Arnsten 2006). Key neural deficits include dysregulation within the prefronto‐parietal attention network and an imbalance in cortical excitation and inhibition (Arnsten 2006). Furthermore, disruptions in noradrenergic and dopaminergic neurotransmission have been implicated in the disorder (Arnsten 2006). These neurobiological abnormalities contribute to impaired executive functioning in affected individuals (Seidman et al. 2005).

Executive functions refer to a set of higher‐order cognitive processes that are essential for goal‐directed behavior (Castellanos et al. 2006). Core components include inhibitory control, working memory, and cognitive flexibility (Seidman et al. 2005). Among these, inhibitory control defined as the capacity to suppress irrelevant or distracting stimuli while maintaining attention on goal‐relevant information has been shown to be particularly compromised in adolescents with ADHD (Castellanos et al. 2006; Fosco et al. 2019). Generally, inhibitory control includes three subdomains: interference control, attentional inhibition, and motor inhibition (Li et al. 2021). Deficits in inhibitory control may adversely impact peer relationships, academic achievement, and overall daily functioning in this population (Fosco et al. 2019).

Growing evidence shows acute aerobic exercise enhances executive function, including inhibitory control, in adolescents with ADHD (Suarez‐Manzano et al. 2018). Proposed mechanisms include exercise‐induced neuroplasticity and neurotransmitter changes (Gomez‐Pinilla and Hillman 2013; Meeusen and De Meirleir 1995; Heijnen et al. 2015). Specifically, three prior studies from our group used controlled crossover designs and transcranial magnetic stimulation (TMS) to demonstrate that acute aerobic exercise improves motor inhibition and increases cortical inhibition in adolescents and young adults with ADHD (Kuo et al. 2024; Kuo et al. 2024; Kuo et al. 2023). Despite these findings, the chronic effects of aerobic exercise on motor inhibition and cortical excitability in this population remain insufficiently explored. To address this gap, the present study aimed to examine both the acute and chronic effects of aerobic exercise on motor inhibition and cortical excitability in adolescents with ADHD. Based on prior research, it was hypothesized that both acute and chronic aerobic exercise would result in significant improvements in motor inhibition and increased cortical inhibition relative to a control intervention. Animal studies showing chronic aerobic exercise modulates dopamine, noradrenaline, and GABAergic systems for days post‐training, alongside human evidence that chronic exercise enhances cortical excitability and executive function (Heijnen et al. 2015; Jiang et al. 2025; Xu et al. 2026). These cumulative mechanisms likely underlie our hypothesis that chronic aerobic exercise would yield greater motor inhibition and cortical inhibition improvements than acute exercise in adolescents with ADHD.

## 2 Methods

### 2.1 Participants

Participants were recruited through flyers, posters, and referrals from the Department of Psychiatry at National Taiwan University Hospital. Inclusion criteria were as follows: (1) adolescents aged 13–17 years with a clinical diagnosis of ADHD according to the *Diagnostic and Statistical Manual of Mental Disorders, Fifth Edition* (DSM‐5), confirmed within the past year by a board‐certified psychiatrist; (2) right‐handedness; and (3) absence of medical contraindications for engaging in aerobic exercise. Exclusion criteria included: presence of any psychiatric, neurological, or medical disorders other than ADHD; history of epilepsy; presence of a cardiac pacemaker; pregnancy; prior surgeries involving cranial implants (e.g., brain electrodes, cochlear implants, aneurysm clips); and concurrent use of medications unrelated to ADHD. Participants who had received behavioral or psychosocial interventions within 1 month prior to and during the study were also excluded. ADHD medications were limited to short‐acting methylphenidate (MPH), which participants were instructed not to take within 12 h to minimize acute pharmacological effects. A power analysis (G*Power 3.1) for a repeated measures ANOVA approach with the within‐subject factors (intervention condition, time course, and ISI), a medium effect size, a critical alpha error of 0.05, and a power of 0.8 resulted in a sample size of 20 participants. Considering the drop‐out rates from previous studies, a total of 24 adolescents with ADHD (15 males and 9 females; mean age = 15.1 ± 1.9 years) was enrolled. Among them, 18 were diagnosed with the combined type, 4 with the inattentive type, and 2 with the hyperactive‐impulsive type. To isolate exercise intervention effects from external physical activity, all participants completed the International Physical Activity Questionnaire (IPAQ) (Craig et al. 2003) at baseline and were instructed to maintain their pre‐study activity levels throughout the experiment (no new sports/exercise programs). Written informed consent was obtained from all participants and their legal guardians. This study was approved by the Research Ethics Committee of National Taiwan University Hospital and conducted in accordance with the Declaration of Helsinki. Demographic data are summarized in Table 1.

**TABLE 1:** Demographic characteristics of the participants.

Characteristics	*n* = 24
Gender (male/female)	15/9
Age (years)	16.1 (3.2)
BMI	23.2 (2.8)
PA score	4019 (1021)

*Note*: PA: physical activity level assessed by the International Physical Activity Questionnaire, expressed in metabolic equivalents of task (MET)‐minutes/week. All data are presented as the means (SD: standard deviation in brackets).

### 2.2 Aerobic Exercise Intervention

The aerobic exercise protocol consisted of a 30‐min cycling session on a stationary bike. Heart rate (HR) and rate of perceived exertion (RPE) were recorded at rest (pre‐intervention), during, and immediately after exercise. HR was continuously monitored using a Polar H7 chest strap (Polar, Finland), and RPE was assessed using the Borg scale (Borg 1982). Each session included a 5‐min warm‐up, 20‐min main session at moderate intensity, and a 5‐min cool‐down. Participants exercised at 50%–70% of their heart rate reserve (HRR), calculated as follows: Maximal HR = 206.9 − (0.67 × age); Target HR = ((Maximal HR − Resting HR) × 0.50–0.70) + Resting HR.

This protocol has been validated for improving executive function in youth with ADHD (Piepmeier et al. 2015; Ludyga et al. 2020; Tsai et al. 2021). HR‐post was assessed within 30 s of completing the exercise protocol.

In the control condition, participants sat on the exercise bike for 30 min while watching a neutral nature documentary. HR was monitored throughout all sessions.

### 2.3 Stop‐Signal Task: Inhibitory Control Assessment

Inhibitory control was evaluated using a stop‐signal task (SST) (Smith et al. 2013). Participants responded to left‐ or right‐pointing arrows (go signal; duration = 250 ms) by pressing the corresponding key. The task consisted of 16 practice trials and 64 experimental trials divided into four blocks. In 25% of the trials (randomly presented), an auditory stop signal (40 ms, 70 dB beep) was presented, indicating the need to inhibit a response. Inter‐trial intervals were 4250 ms (practice) and 2250 ms (experimental). Outcome measures included stop‐signal reaction time (SSRT), go reaction time (go RT), stop accuracy (correct inhibitions), and commission errors (failed inhibitions). Parallel versions of the task were used across sessions and were randomized in order.

### 2.4 TMS: Cortical Excitability Assessment

Cortical excitability was assessed via TMS using a figure‐of‐eight coil (70 mm) connected to a Magstim 200 stimulator (Magstim, UK). TMS generates a strong, short‐lasting magnetic stimulus that induces an electric current in the motor cortex, allowing activation of neurons at a suprathreshold level to elicit motor evoked potentials (MEPs) in the targeted muscle. Surface electromyography (EMG) was recorded from the right abductor digiti minimi (ADM) muscle using Ag/AgCl electrodes. SI_1mv_ was defined as the minimum TMS output required to evoke stable MEPs with an amplitude of about 1 mV. To minimize motion artifacts, participants were instructed to fully relax the target muscle and forearm and were continuously monitored online. Trials contaminated by motion artifact, coil displacement, or excessive background EMG were excluded. Exclusion was performed visually offline, blinded to condition; trials showing a deviation above or below baseline within the 50 ms preceding the TMS pulse were removed. Resting motor threshold (RMT) was determined as the minimum TMS output required to evoke reliable MEPs with an amplitude of approximately 50–100 µV in at least 5 out of 10 consecutive stimuli delivered to the relaxed muscle (Rossi et al. 2021). Active motor threshold (AMT) was defined as the minimum TMS intensity needed to generate an MEP exceeding the baseline spontaneous muscle activity (approximately 15% of maximum muscle strength) in at least 5 out of 10 consecutive trials (Rossi et al. 2021). These thresholds primarily reflect neuronal membrane excitability and are modulated by sodium‐channel activity, they can be enhanced by voltage‐gated sodium channel blockers but are not significantly affected by substances that influence gamma‐aminobutyric acid (GABA)‐ergic or glutamatergic transmission (Ziemann et al. 1996). Paired‐pulse TMS was used to evaluate short‐interval intracortical inhibition (SICI) and intracortical facilitation (ICF). A conditioning pulse (70% AMT) preceded a test pulse adjusted to elicit 1 mV MEPs (SI1mV). Interstimulus intervals (ISIs) of 2, 3, 5, 10, and 15 ms were employed to evaluate SICI and ICF (Ilić et al. 2002). Here, 20 trials per ISI were recorded. The order of single‐ and paired‐pulse trials, as well as the ISIs, was presented in a pseudorandomized sequence to prevent order and habituation effects. Typically, the first three ISIs (2, 3, and 5 ms) induce inhibitory effects, while the last two ISIs (10 and 15 ms) promote facilitatory effects, reflecting the excitability of inhibitory and excitatory interneurons (Ziemann et al. 1998). SICI is primarily mediated by GABAergic neurons, although it also reflects the activity of glutamatergic neurons to a lesser extent (Ziemann et al. 1996). ICF is mainly governed by glutamate, but it can also be influenced by GABA (Ziemann et al. 1996).

### 2.5 Experimental Design

**FIGURE 1:**
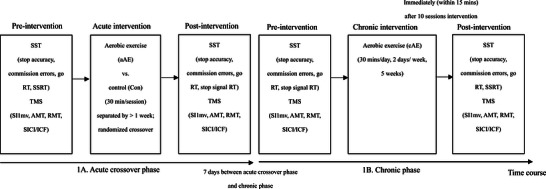
Experimental course of the study. The study was conducted in two phases (1A: acute and 1B: chronic). For the acute phase, participants received a single bout of aerobic exercise (aAE) and a control intervention (Con). For the chronic phase, the same participants took part in the chronic aerobic exercise (cAE) intervention (30 min/day, 2 days/week, 5 weeks). SST (inhibitory control performance) and TMS measurements (cortical excitability) were assessed before and after interventions (Con, aAE, and cAE). A questionnaire‐based interview (asking whether the participants felt fatigue, headache, or dizziness) was conducted after the final assessments for acute and chronic aerobic exercise intervention.

The study used a within‐subject, partial cross‐over design with both acute and chronic interventions (Figure 1). All participants first completed the acute phase, consisting of a single acute aerobic exercise (aAE) session and a control (Con) session in randomized counterbalanced order, separated by at least 7 days to prevent carry‐over effects. After the acute phase (mean interval: 14.2 ± 3.1 days), participants underwent the chronic phase (chronic aerobic exercise: cAE), which included 10 sessions of aerobic exercise (2 times/week for 5 weeks). Unlike the acute phase, the chronic intervention was not randomized and was thus more susceptible to time‐ and order‐related influences. Pre‐ and post‐intervention assessments (SST and TMS) were conducted for each condition (aAE_pre/aAE_post, Con_pre/Con_post, and cAE_pre/cAE_post). This sequential design minimized interference between phases while maximizing statistical power via within‐subject comparisons. Participants maintained consistent external physical activity levels, verified weekly via IPAQ, throughout both phases. The cAE_pre assessment occurred before the first chronic session, and cAE_post was performed immediately (within around 15 min) after the 10th session. During TMS assessments, participants sat comfortably while SI1mV, RMT, AMT, and SICI/ICF were measured using established procedures (as described above). A 15‐min rest followed AMT determination to minimize confounding effects from muscle contraction. After each session (acute and chronic phases), participants were screened for adverse effects such as fatigue, headache, or dizziness via structured questionnaires.

### 2.6 Data Analysis

Aerobic exercise variables (RPE; HR‐pre: heart rate before intervention; HR‐avg: heart rate during intervention; HR‐post: heart rate after intervention) were analyzed using repeated‐measures ANOVA with intervention condition (Con, aAE, and cAE) as the within‐subject factor. To reduce baseline bias from session order, pre‐intervention measures (SST and TMS outcomes) were compared across all three conditions (Con_pre, aAE_pre, and cAE_pre). Given the sample size and primary focus on within‐subject pre–post changes, we analyzed all three conditions (Con, aAE, and cAE) in a single repeated‐measures ANOVA, rather than a formal two‐sequence cross‐over model with sequence effects for the acute versus control conditions. Thus, the chronic condition should be interpreted as an additional within‐subject condition, not a third cross‐over period.

For inhibitory control performance, repeated measures ANOVAs were performed with the outcome measures of the SST (stop accuracy, commission errors, go RT, and SSRT) as dependent variables, and with the within‐subject factors intervention condition (Con, aAE, and cAE), and time course (pre‐ and post‐intervention).

Cortical excitability measures (SI_1mV_, RMT, and AMT) were analyzed as means of TMS intensity before and after acute/chronic aerobic exercise and control interventions. EMG data were exported and analyzed offline in MATLAB, with MEPs quantified as peak‐to‐peak amplitude in a 15–50 ms window post‐TMS pulse. An investigator blinded to condition (Con, aAE, and cAE) and time point (pre and post) performed all offline EMG and MEP analyses. Repeated‐measures ANOVAs used these parameters (SI_1mV_, RMT, and AMT) as dependent variables, with intervention condition (Con, aAE, and cAE) and time (pre and post) as within‐subject factors. For SICI/ICF, paired‐pulse MEPs were normalized to the mean single‐pulse SI_1mV_ amplitude from the same block; mean normalized amplitudes for each ISI (2, 3, 5, 10, and 15 ms), time point, and condition served as the dependent variable in repeated‐measures ANOVAs. Mauchly's test assessed sphericity, with Greenhouse–Geisser corrections applied when violated. Post hoc tests used Bonferroni‐corrected Student's *t*‐tests. Effect sizes were reported as partial eta‐squared ($\eta_{p}^{2}$).

Simple bivariate correlations (Pearson's *r* coefficient) were performed to investigate the relationships between baseline inhibitory control performance (SSRT), and cortical excitability parameters (SI_1mV_, RMT, AMT, SICI: MEP means of ISI 2, 3, and 5 ms, and ICF: means of ISI 10 and 15 ms). To explore the impact of aerobic exercise on the association between inhibitory control performance and cortical excitability, simple bivariate correlations were conducted to investigate the relationships between the changes in inhibitory control performance (SSRT) (aAE_post_aAE_pre; cAE_post_cAE_pre; Con_post_Con_pre) and the neurophysiological parameters (SI_1mv_, RMT, AMT, SICI: MEP means of the ISI 2,3, and 5 ms, and ICF: means of ISI 10 and 15 ms) under the aerobic exercise (aAE_post_aAE_pre; cAE_post_cAE_pre) and control interventions (Con_post_Con_pre). All statistical analyses were conducted using IBM SPSS Statistics, version 22.

## 3 Results

All 24 enrolled participants tolerated the protocols well, completing all six assessment sessions (Con_pre, Con_post, aAE_pre, aAE_post, cAE_pre, and cAE_post) with no dropouts or missing SST/TMS data. Based on a structured questionnaire‐based interview, none of the subjects reported any adverse effects related to the intervention. Descriptive data for RPE and HR parameters are presented in Table 2. Repeated‐measures ANOVA revealed significant differences in HR‐avg across conditions. Both aAE and cAE conditions showed significantly higher HR‐avg compared to the Con condition. However, no significant differences were observed among intervention conditions for RPE, HR‐pre, or HR‐post. Furthermore, there were no significant differences for baseline SST (SSRT, go RT, stop accuracy, and commission errors) or TMS (SI_1mV,_ AMT, RMT, and SICI/ICF) outcomes for pre‐intervention measures across all three conditions (Con_pre, aAE_pre, cAE_pre).

**TABLE 2:** Comparison of rate of perceived exertion (RPE), and heart rate (HR) (pre, average, and post) in relation to intervention conditions (control [Con], acute aerobic exercise [aAE], and chronic aerobic exercise [cAE]).

Variable	Con	aAE	cAE	df	*F* value	*p*
RPE	N/A	13.8 (1.5)	13.4 (2.2)	2	0.702	0.504
HR‐pre (bpm)	83.2 (8.1)	82.5 (9.2)	82.8 (8.2)	2	0.744	0.403
HR‐avg (bpm)	83.4 (9.1)	135.2 (8.4)	134.3 (8.3)	2	9.609	**0.008**
HR‐post (bpm)	84.4 (7.1)	88.3 (7.6)	87.8 (7.3)	2	0.427	0.524

*Note*: HR‐pre: df, degrees of freedom. The data are presented as the means (SD: standard deviation); heart rate before intervention; HR‐avg: heart rate during intervention; HR‐post: heart rate after intervention.

### 3.1 Inhibitory Control Performance (SST)

**FIGURE 2:**
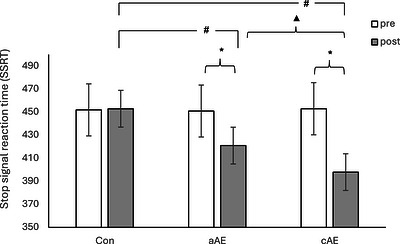
Stop signal reaction time (SSRT) was assessed before and after intervention (control [Con], acute aerobic exercise [aAE], and chronic aerobic exercise [cAE]). Before intervention, there was no significant difference between baseline conditions. After intervention, acute and chronic aerobic exercise significantly reduced RT compared to baseline, respectively. Furthermore, acute and chronic AE significantly reduced RT compared to the control condition. Asterisks indicate significant differences of RT before and after the intervention (aAE_pre vs. aAE_post; cAE_pre vs. cAE_post). Hash symbols represent significant differences of RT after acute and chronic AE versus the control intervention (aAE_post vs. control_post; cAE_post vs. control_post). The triangle implies significant differences of RT between cAE_post and aAE_post. Vertical bars depict the standard error of the mean (SEM).

As shown in Table 3 and Figure 2, repeated‐measures ANOVA revealed significant main effects for intervention condition (*F*[2] = 11.072, *p* = 0.005) and time (F[1] = 8.092, *p* = 0.013), as well as a significant intervention × time interaction (F[2] = 14.653, *p* = 0.002). Post hoc analysis indicated that SSRT significantly decreased following chronic aerobic exercise (cAE_post), relative to both acute aerobic exercise (aAE_post) and control (Con_post) conditions. No significant differences in SSRT were found among conditions at the pre‐intervention stage. Within‐condition comparisons further demonstrated a significant decrease in SSRT from pre‐ to post‐intervention for both aAE and cAE, while no changes were observed in the control condition. No differences were found for the other outcome measures (stop accuracy, commission errors, and go RT).

**TABLE 3:** Repeated‐measures ANOVA results of stop signal task (SST) performance (inhibitory control).

Parameters	Conditions	df	*F* value	*p* value	*η* ^2^ *_p_*
Stop accuracy	Intervention	2	0.480	0.5	0.033
	Time	1	0.086	0.774	0.006
	Intervention × time	2	4.166	0.061	0.09
Commission errors	Intervention	2	1.190	0.294	0.078
	Time	1	0.059	0.812	0.004
	Intervention × time	1	2.687	0.123	0.03
Go RT	Intervention	2	2.281	0.153	0.14
	Time	1	2.281	0.153	0.14
	Intervention × time	2	4.054	0.064	0.12
SSRT	Intervention	2	11.072	**0.005**	0.443
	Time	1	8.092	**0.013**	0.366
	Intervention × time	2	14.653	**0.002**	0.511

*Note*: Significant results are shown in bold (*p* < 0.05).

Abbreviation: RT: reaction time.

### 3.2 Cortical Excitability (TMS)

**FIGURE 3:**
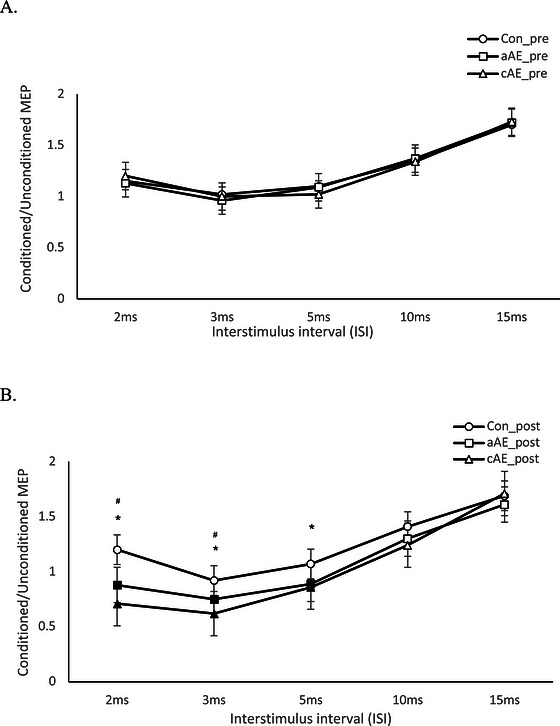
Short‐interval intracortical inhibition and intracortical facilitation (A) before and (B) after intervention (control [Con], acute aerobic exercise [aAE], and chronic aerobic exercise [cAE]). Single pulse‐standardized double pulse stimulation MEP amplitude ratios ± SEM are depicted for ISIs with inhibitory (ISIs of 2, 3, and 5 ms) and facilitatory (ISIs of 10 and 15 ms) intervals for the different interventions. Before intervention, no significant differences between baseline conditions were observed. After intervention, aAE_post and cAE_post conditions show decreased intracortical excitability. In the post_aAE condition, inhibition was significantly increased at ISIs 2, 3, and 5 ms, as compared to aAE_pre, and control_post conditions. In the cAE_post condition, inhibition was significantly increased at the ISIs of 2, 3, and 5 ms compared to the cAE_pre condition and control_post conditions. Furthermore, in the cAE_post condition inhibition was significantly increased at the ISIs of 2 ms, and 3 ms compared to the aAE_post condition. Filled symbols indicate significant differences between the respective AE_pre versus AE_post interventions. Asterisks indicate significant differences after aAE and cAE compared to the control condition for respective ISIs. The hash symbol represents significant differences after aAE and cAE interventions (aAE_post vs. cAE_post). Vertical bars depict the standard error of the mean (SEM).

As summarized in Table 4, repeated‐measures ANOVA for SI_1mV_, RMT, and AMT revealed no significant main effects or interactions for intervention condition or time (*p* > 0.05). In contrast, for SICI and ICF, the analysis yielded significant main effects of intervention condition (*F*[2] = 6.067, *p* = 0.01), time (F[1] = 10.623, *p* = 0.003), and ISI (F[4] = 5.547, *p* = 0.021), along with significant two‐ and three‐way interactions: intervention × time: F(2) = 9.247, *p* = 0.002, intervention × ISI: F(8) = 9.606, *p* = 0.008, time × ISI: F(4) = 6.767, *p* = 0.021, intervention × time × ISI: F(8) = 11.602, *p* = 0.004). Post hoc analyses revealed that both acute and chronic aerobic exercise significantly increased intracortical inhibition at ISIs of 2, 3, and 5 ms compared to the control condition post‐intervention. Furthermore, cAE_post exhibited significantly greater inhibition at ISIs of 2 and 3 ms than aAE_post. No significant differences in SICI were observed across conditions at baseline. Within‐condition analyses showed significant increases in inhibition at ISIs 2, 3, and 5 ms following aAE and cAE, but not following the control intervention (Figure 3, Table S1).

**TABLE 4:** Repeated‐measures ANOVA results of transcranial magnetic stimulation (TMS) (cortical excitability).

Parameters	Conditions	df	*F* value	*p* value	*η* ^2^ *_p_*
SI_1mv_	Intervention	2	4.168	0.11	0.16
	Time	1	2.523	0.228	0.10
	Intervention × time	2	0.997	0.466	0.04
AMT	Intervention	2	0.867	0.405	0.07
	Time	1	2.72	0.174	0.11
	Intervention × time	2	0.321	0.601	0.01
RMT	Intervention	2	1.507	0.278	0.06
	Time	1	0.054	0.948	0.01
	Intervention × time	2	0.702	0.504	0.03
SICI‐ICF	Intervention	2	6.067	**0.01**	0.3
	Time	1	10.623	**0.003**	0.43
	ISI	4	5.547	**0.021**	0.28
	Intervention × time	2	9.247	**0.002**	0.4
	Intervention × ISI	8	9.606	**0.008**	0.41
	Time × ISI	4	6.767	**0.021**	0.33
	Intervention × time × ISI	8	11.602	**0.004**	0.45

*Note*: Significant results are shown in bold (*p* < 0.05).

Abbreviations: RMT: AMT: active motor threshold; resting motor threshold; SICI‐ICF: short‐interval intracortical inhibition and facilitation.

**FIGURE 4:**
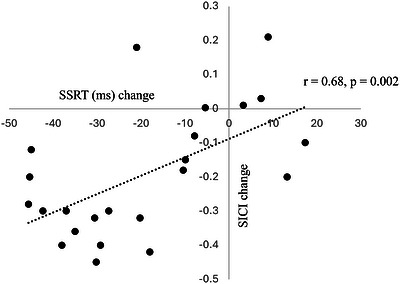
Significant positive correlations were observed between changes in cortical inhibition (TMS: SICI) (mean over inhibitory ISIs) and changes in inhibitory control (SST: SSRT) under chronic aerobic exercise condition (*r* = 0.68, *p* = 0.002).

At baseline, SICI showed a significant positive correlation with SSRT across all conditions, indicating that reduced cortical inhibition was associated with slower stopping performance. No significant associations were observed between SSRT and SI_1mV,_ RMT, AMT, or ICF. Likewise, changes in SSRT were not significantly correlated with changes in SI_1mV_, RMT, or AMT in any condition. In contrast, changes in SICI, averaged across inhibitory ISIs, were significantly correlated with changes in SSRT under both acute aerobic exercise (*r* = 0.65, *p* = 0.004) and chronic aerobic exercise (*r* = 0.68, *p* = 0.002) (Figure 4), whereas no significant correlation was found in the control condition. These results indicate that greater increases in cortical inhibition were associated with better inhibitory control. Changes in ICF, averaged across facilitatory ISIs, were not significantly associated with changes in SSRT in any condition.

## 4 Discussion

### 4.1 Behavioral‐Cortical Correlation

This study investigated the effects of acute and chronic aerobic exercise on inhibitory control and cortical excitability in adolescents with ADHD. At baseline, no significant differences were observed between intervention conditions (Con, aAE, and cAE) in either inhibitory control performance or cortical excitability measures. Importantly, a significant positive correlation was found between SICI and SSRT, indicating that lower intracortical inhibition was associated with poorer inhibitory control at baseline. Both acute and chronic aerobic exercise interventions led to significant improvements in inhibitory control. These behavioral effects were paralleled by physiological changes, as demonstrated by increased intracortical inhibition following both exercise interventions. Notably, the chronic intervention elicited a more pronounced enhancement of cortical inhibition than the acute protocol. Furthermore, the increase in cortical inhibition was significantly correlated with improvements in inhibitory control, suggesting a mechanistic link between exercise‐induced cortical excitability and inhibitory control function enhancements in adolescents with ADHD.

### 4.2 Differential Mechanisms of Acute Versus Chronic Aerobic Exercise

At baseline, poorer motor inhibition (longer SSRT) was associated with reduced SICI, confirming impaired GABAergic inhibition as a core neurophysiological deficit in adolescents with ADHD (Puts et al. 2020; Ende et al. 2016). Previous studies have reported that acute moderate‐intensity aerobic exercise can improve motor inhibition in this population (Suarez‐Manzano et al. 2018; Kuo et al. 2024). The present findings extend this evidence by demonstrating that not only acute, but also chronic aerobic exercise significantly enhances motor inhibition in adolescents with ADHD. Both acute and chronic aerobic exercise improved SSRT, paralleled by increased SICI at ISIs 2, 3, and 5 ms; however, effects absent in the control condition.

Chronic exercise yielded superior inhibition specifically at ISIs 2 and 3 ms (cAE_post > aAE_post), suggesting cumulative GABAergic plasticity beyond single‐session transients. This pattern aligns with animal data showing repeated aerobic bouts upregulate GABAergic receptors, producing more stable intracortical inhibition (Moll et al. 2000). The greater chronic SSRT improvement further supports training‐induced executive function gains outpacing acute effects.

### 4.3 Comparison With Pharmacological Mechanisms

Previous research has demonstrated that pharmacological enhancement of dopamine via MPH increases SICI and improves cognitive performance in children with ADHD (Moll et al. 2000). These findings align with prior work from our group, which reported a positive association between aerobic exercise‐induced increases in cortical inhibition and improvements in motor inhibition in adolescents with ADHD. Thus, exercise‐induced dopaminergic modulation may represent another pathway through which aerobic exercise enhances inhibitory control in this population. These parallel mechanisms position aerobic exercise as a viable non‐pharmacological adjunct, particularly given MPH's side effect profile and variable long‐term efficacy in adolescents.

Despite these current findings, several limitations of this study warrant consideration. First, due to the small sample size, baseline characteristics, such as physical condition, fitness and activity levels were not explored as possible confounders. Another limitation concerns the heterogeneity of ADHD subtypes in our sample. Most participants were diagnosed with the combined type (*n* = 18), whereas only a few had the inattentive (*n* = 4) or hyperactive‐impulsive (*n* = 2) presentations. As a result, the present findings largely reflect adolescents with combined‐type ADHD, and our data do not allow reliable subtype‐specific inferences regarding cortical excitability or exercise responsiveness. Exploratory sensitivity checks excluding the two hyperactive‐impulsive cases did not alter the pattern or significance of the main intervention effects on SSRT or SICI/ICF, but these analyses are underpowered and should be interpreted with caution. Future studies with larger and more balanced subtype samples are needed to determine whether different ADHD presentations show distinct neurophysiological responses to aerobic exercise. Second, as the participants were allowed to continue with their physical activity routines, the results might have been affected by chronic exercise or fatigue. The study checked the participants’ physical activity levels every week to ensure that the activity level remained similar throughout the experiment. Weekly IPAQ assessments (telephone/email) during the chronic phase verified adherence, with mean MET‐min/week remaining stable (baseline: 4019 ± 1021; Week 5: 4052 ± 987; *p* = 0.82). Participants exceeding ±20% baseline deviation were excluded from analysis (*n* = 0). This rigorous monitoring ensured that observed SSRT/SICI changes reflected the protocol‐specific exercise (aAE/cAE) rather than uncontrolled fitness gains. However, a rigorous or even moderate physical activity regimen should be applied in the future study. Third, an important methodological limitation is that the chronic aerobic exercise intervention was always delivered after the acute and control sessions, and no chronic control intervention was included. Although baseline behavioral and TMS measures did not differ among Con_pre, aAE_pre, and cAE_pre, we cannot fully exclude sequence and time effects (e.g., task practice, increasing familiarity with the equipment, or developmental/maturational changes) as contributors to the observed chronic effects. The chronic findings may be partly driven by an acute response to the last session and should therefore be interpreted with caution. Therefore, the chronic intervention findings should be regarded as more exploratory and interpreted with caution until replicated in a fully randomized cross‐over or parallel‐group design including an appropriate chronic control condition. Furthermore, the total dose of the chronic exercise program was relatively low: participants engaged in 30‐min sessions (including warm‐up and cool‐down) twice per week for 5 weeks. Although this schedule achieved moderate aerobic intensity in adolescents, the overall intervention dose was modest. Meta‐analytic work in children and adolescents with ADHD indicates that such shorter, lower‐dose programs can produce small‐to‐moderate improvements in inhibitory control (Dastamooz et al. 2023; Lambez et al. 2020), whereas longer interventions often yield larger effects. Accordingly, the present chronic effects should be viewed as modest, likely reflecting the combination of repeated acute benefits and early training‐related adaptations, and future studies using higher total doses are needed to determine the full potential of exercise as an adjunctive treatment in this population. Fourth, the mechanistic interpretations are preliminary, as TMS measures are not receptor‐specific and were not recorded in regions directly involved in executive functions. Future studies employing neurotransmitter‐specific techniques such as magnetic resonance spectroscopy (MRS), functional MRI (fMRI), PET, or TMS‐EEG may elucidate the underlying mechanisms more specifically. In addition, the absence of a healthy control group limits the findings. Future work should include such a comparison group and control for these factors to more precisely determine ADHD‐specific effects. Moreover, potential confounding effects of familiarity, practice, and developmental changes cannot be ruled out. As such, the findings must be interpreted with caution. Lastly, the lack of long‐term follow‐up data precludes conclusions regarding the durability of the observed effects. Future studies should assess whether the benefits of aerobic exercise persist over time.

## 5 Conclusions

This study demonstrates that chronic aerobic exercise produces superior enhancements in cortical inhibition and motor inhibition compared to acute aerobic exercise in adolescents with ADHD. The strong correlation between SICI and SSRT changes establishes a neurophysiological mechanism linking aerobic exercise to executive function improvement. These GABAergic effects parallel MPH's action but through sustained neuroplasticity rather than acute pharmacology. Regular moderate‐intensity aerobic exercise, which is feasible even at low doses (2 times/week), emerges as a promising non‐pharmacological strategy to strengthen inhibitory control in this population.

## Author Contributions

**Jia‐Ling Sun**: investigation, writing – original draft, methodology, formal analysis, project administration, data curation, writing – review and editing. **Hsiao‐I Kuo**: conceptualization, investigation, funding acquisition, writing – original draft, writing – review and editing, supervision, resources. **Jung‐Chi Chang**: conceptualization, investigation, writing – review and editing, project administration. **Pou‐Leng Cheong**: conceptualization, investigation, writing – review and editing, project administration.

## Funding

The study is supported by the National Science and Technology Council, Taiwan, grant “112‐2314‐B‐002‐119‐MY3”.

## Conflicts of Interest

The authors declare no conflicts of interest.

## Supporting information




**Supplementary Table**: brb371600‐sup‐0001‐TableS1.docx

## Data Availability

The data that support the findings of this study are available from the corresponding author upon reasonable request.
