# Supplementary material for: Acute and Chronic Effects of Aerobic Exercise on Motor Inhibition and Cortical Excitability in Adolescents With Attention Deficit Hyperactivity Disorder
Source: Brain Behav. 2026 Aug 5;16(8):e71600. doi: 10.1002/brb3.71600 (PMC13439208; doi:10.1002/brb3.71600)
Supplement: Supplementary file 1 [file BRB3-16-e71600-s001.docx]

Supplementary

Table 1. Mean and standard deviations (SD) of the cortical excitability parameters of pre- and post-acute/chronic aerobic exercise and control intervention.

| Acute  aerobic exercise | | | | Chronic  Aerobic exercise | | | Control | | |
| --- | --- | --- | --- | --- | --- | --- | --- | --- | --- |
| Variable | Pre | Post | P | Pre | Post | P | Pre | Post | P |
| 2ms | 1.13 (0.2) | 0.88 (0.16) | **0.001** | 1.2 (0.22) | 0.71 (0.12) | **0.02** | 1.15 (0.22) | 1.2 (0.1) | 0.41 |
| 3ms | 0.96 (0.1) | 0.75 (0.2) | **0.01** | 1.0 (0.12) | 0.62 (0.2) | **0.03** | 1.02 (0.12) | 0.92 (0.13) | 0.32 |
| 5ms | 1.09 (0.22) | 0.89 (0.1) | **0.03** | 1.02 (0.23) | 0.86 (0.14) | **0.001** | 1.1 (0.13) | 1.07 (0.22) | 0.67 |
| 10ms | 1.37 (0.21) | 1.3 (0.11) | 0.39 | 1.34 (0.2) | 1.24 (0.28) | 0.62 | 1.35 (0.14) | 1.41 (0.19) | 0.71 |
| 15ms | 1.72 (0.19) | 1.61(0.28) | 0.32 | 1.73 (0.1) | 1.57 (0.18) | 0.58 | 1.7 (0.27) | 1.69 (0.23) | 0.88 |

All data are presented as the means (SD: standard deviation in brackets).
